# Genetic characterizations of *Cryptosporidium* spp. from children with or without diarrhea in Wenzhou, China: high probability of zoonotic transmission

**DOI:** 10.1186/s12866-024-03273-w

**Published:** 2024-04-04

**Authors:** Wei Zhao, Guangxu Ren, Weiyan Jiang, Long Wang, Jiayang Wang, Zhongying Yuan, Lanzhu Yan, Yongtai Li, Yanbin Sun, Xinjie Xue, Yanyan Jiang, Gang Lu, Huicong Huang

**Affiliations:** 1 Department of Parasitology, School of Basic Medical Sciences, Wenzhou Medical University, Wenzhou, Zhejiang 325035 China; 2 Department of Pathogenic Biology, Hainan Medical University, Haikou, Hainan China; 3 Hainan Medical University-The University of Hong Kong Joint Laboratory of Tropical Infectious Diseases, Hainan Medical University, Haikou, Hainan China; 4 Key Laboratory of Tropical Translational Medicine of Ministry of Education, Hainan Medical University, Haikou, 571199 China; 5 The Second School of Medical, Affiliated Hospital and Yuying Children’s Hospital, Wenzhou Medical University, Wenzhou, Zhejiang China; 6 National Institute of Parasitic Diseases, Chinese Center for Disease Control and Prevention (Chinese Center for Tropical Diseases Research), NHC Key Laboratory of Parasite and Vector Biology, WHO Collaborating Centre for Tropical Diseases, National Center for International Research on Tropical Diseases, Shanghai, 200025 China; 7 Department of Laboratory and Pathology, Hebei Provincial Corps Hospital of Chinese People’s Armed Police Force, Shijiazhuang, Hebei 050081 China

**Keywords:** *Cryptosporidium*, Children, Genetic characteristics, China

## Abstract

**Background:**

*Cryptosporidium* is a highly pathogenic parasite responsible for diarrhea in children worldwide. Here, the epidemiological status and genetic characteristics of *Cryptosporidium* in children with or without diarrhea were investigated with tracking of potential sources in Wenzhou City, China.

**Methods:**

A total of 1032 children were recruited, 684 of whom had diarrhea and 348 without, from Yuying Children’s Hospital in Wenzhou, China. Samples of stool were collected from each participant, followed by extraction of DNA, genotyping, and molecular identification of *Cryptosporidium* species and subtypes.

**Results:**

Twenty-two of the 1032 (2.1%) children were infected with *Cryptosporidium spp*. with 2.5% (17/684) and 1.4% (5/348) in diarrhoeic and asymptomatic children, respectively. Four *Cryptosporidium* species were identified, including *C. parvum* (68.2%; 15/22), *C. felis* (13.6%; 3/22), *C. viatorum* (9.1%; 2/22), and *C. baileyi* (9.1%; 2/22). Two *C. parvum* subtypes named IIdA19G1 (*n* = 14) and IInA10 (*n* = 1), and one each of *C. felis* (XIXa) and *C. viatorum* (XVaA3g) subtype was found as well.

**Conclusions:**

This is the first research that identified *Cryptosporidium* in children of Wenzhou, China, using PCR. Identification of zoonotic *C. parvum, C. felis*, *C. viatorum*, and their subtypes indicate potential cross-species transmission of *Cryptosporidium* between children and animals. Additionally, the presence of *C. baileyi* in children suggests that this species has a wider host range than previously believed and that it possesses the capacity to infect humans.

## Background

*Cryptosporidium* is a class of protozoan parasite with the ability to infect epithelial cells of the gastrointestinal tract of vertebrate species [1]. Cryptosporidiosis is often a self-limiting infection in individuals with a fully functioning immune system, but it can lead to severe diarrhea and potentially cause death in individuals with Acquired Immunodeficiency Syndrome (AIDS), infants, and other immunocompromised or deficient individuals [2]. Cryptosporidiosis is a significant contributor to mortality associated with moderate to severe diarrhea in children residing in low- and middle-income countries and represents an important but often underestimated public health threat in developed countries [3].

*Cryptosporidium* can infect a diverse range of animal species and is distributed globally. The zoonotic transmission of diseases from animals to humans is seen as a significant concern for public health [4]. The infectious stage of *Cryptosporidium*, known as the oocyst, favors direct transmission since it becomes infectious as soon as it is excreted with the host’s feces [5]. Oocysts can endure the surrounding environment and resist conventional water treatment methods. They may be transferred to people either by interacting directly with animals or their waste products, or by consuming water and food that have been contaminated with oocysts [6]. The examination of sporadic instances and epidemics of cryptosporidiosis has improved the present knowledge of the risk factors and sources of infection.

In recent decades, there have been notable advancements in molecular typing techniques, which have played a crucial role in enhancing the current knowledge of epidemiology regarding cryptosporidiosis across various seasonal, geographical, and socioeconomic contexts [7]. Currently, the most commonly used method for identification of the species is by the small subunit ribosomal RNA (*SSU rRNA*) sequence with both highly variable and conserved sequences [7]. At present, there have been a total of 48 accepted species and more than 120 genotypes of *Cryptosporidium*. Of these, 21 species and two genotypes have been linked to human infection; *C. hominis* and *C. parvum* represent around 95% of human infection cases [5]. Although humans are the main hosts of *C. hominis*, this parasite is also reported in domestic and wild animals, including sheep, goats, and cattle [5, 8]. *C. parvum* mainly infects artiodactyls and humans, but it is also frequently reported in various rodents and wild animals [5]. A number of subtyping instruments based on the glycoprotein 60-kDa (*gp60*) gene were developed to identify the human source of *Cryptosporidium* infection precisely [9–11]. The proposed and widely adopted *gp60* nomenclature system has greatly facilitated the analysis of global epidemiology, as the variability in the sequence of this marker has been instrumental in inferring transmission routes [12]. However, there is still uncertainty regarding the epidemiology of *Cryptosporidium* and its spread in humans, particularly in underdeveloped regions of the world, requiring further research.

A cumulative count of approximately 6,000 human cases of cryptosporidiosis has been documented in 27 provinces of China [13], yet data involving molecular studies remains limited. Out of the total of 68 studies that were undertaken, only 10 focused on the molecular analysis of *Cryptosporidium* species [13–16]. Even though fewer than 300 cases were genetically characterised, a total of nine distinct species of *Cryptosporidium* were detected, including *C. andersoni*, *C. felis, C. parvum, C. hominis*, *C. viatorum, C. meleagridis, C. canis, C. occultus*, and *C. suis* [13–16]. The isolates were subjected to further classification into subtypes by the utilization of sequence analysis of the *gp60* gene, including fifteen *C. hominis* subtypes, three *C. parvum* subtypes, four *C. meleagridis* subtypes, and one *C. viatorum* subtype [13–16]. However, molecular data from many regions, such as Wenzhou, remain unavailable. This study is the first baseline epidemiological study of human *Cryptosporidium* in Wenzhou, focusing on exploring the characteristics of *Cryptosporidium* in children with or without diarrhea.

## Results

### Prevalence of *Cryptosporidium*

According to the analysis of the *SSU rRNA* gene, *Cryptosporidium* was identified in 2.1% (22/1032, 95% CI: 1.4–3.2%) of the samples, with 17/684 (2.5%, 95% CI: 1.6-4.0%) in the patients with diarrhea and 5/348 (1.4%, 95% CI: 0.6–3.3%) in the asymptomatic population (Table 1). The prevalence of *Cryptosporidium* in individuals experiencing diarrhea was found to be greater in comparison to the prevalence seen in the asymptomatic group, but there was no statistical difference (χ2 = 1.216; *p* = 0.363). In the group of children suffering from diarrhea, the prevalence was found to be 2.9% (11/376, 95% CI: 1.6–5.1%) among boys and 1.9% (6/308, 95% CI: 0.9–4.2%) among girls. Moreover, in the asymptomatic group, the prevalence of *Cryptosporidium* was 2.1% (4/195, 95% CI: 0.8–5.2%) and 0.7% (1/153, 95% CI: 0.1–3.6%) for boys and girls, respectively. Statistical analysis revealed no significant difference in the prevalence rate of *Cryptosporidium* was observed through paired comparisons between boys and girls (χ2 = 0.667; *p* = 0.414 for diarrhea group and *p* = 0.390 for asymptomatic group).

**Table 1:** Prevalence and species distribution of *Cryptosporidium* among children from Wenzhou of China by counties clinical symptoms and gender

Clinical symptoms	No.Positive/No. Examined (%, 95%CI)	Cryptosporidium species (n)/gp60 subtypes (n)
Diarrhea		
Boy	11/376 (2.9, 1.6–5.1)	*C. parvum* (8)/IIdA19G1 (7), IInA10 (1); *C. viatorum* (1)XVaA3g (1); *C. baileyi* (2)/-
Girl	6/308 (1.9, 0.9–4.2)	*C. parvum* (6)/IIdA19G1 (6)
Subtotal	17/684 (2.5, 1.6-4.0)	*C. parvum* (14)/IIdA19G1 (13), IInA10 (1); *C. viatorum* (1)/XVaA3g (1); *C. baileyi* (2)/-
Non-Diarrhea		
Boy	4/195 (2.1, 0.8–5.2)	*C. parvum* (1)/ IIdA19G1 (1); *C. felis* (2)/XIXa (2), *C. viatorum* (1)/XVaA3g (1)
Girl	1/153 (0.7, 0.1–3.6)	*C. felis* (1)/XIXa (1)
Subtotal	5/348 (1.4, 0.6–3.3)	*C. parvum* (1)/ IIdA19G1 (1); *C. felis* (3)/XIXa (3); *C. viatorum* (1)/XVaA3g (1)
Total	22/1032 (2.1, 1.4–3.2)	*C. parvum* (15)/IIdA19G1 (14), IInA10 (1); *C. viatorum* (2)/XVaA3g (2); *C. felis* (3)/XIXa (3); *C. baileyi* (2)/-

### Genetic characterization based on the *SSU rRNA* gene

The present work involved the investigation of 22 *Cryptosporidium* isolates, whereby the sequencing analysis revealed the presence of four distinct *Cryptosporidium* species, including *C. viatorum, C. parvum*, *C. felis*, and *C. baileyi* (Table 1). Among the examined specimens, it was seen that *C. parvum* exhibited the highest prevalence rate, accounting for 21.3% (15/22). This was followed by *C. felis*, which had a prevalence rate of 10.6% (3/22). Additionally, *C. viatorum* and *C. baileyi* were found to have equal prevalence rates of 2.1% (1/47) each. *C. parvum* and *C. viatorum* were detected among both the diarrhea patients and the asymptomatic population, while *C. felis* was only found in diarrhea patients, and *C. baileyi* was observed only in the asymptomatic individuals (Table 1).

Fifteen isolates of *C. parvum* were represented by seven sequences (OR815985 to OR815991). These sequences exhibited a high level of similarity, ranging from 99.41 to 100% when compared to the published *C. parvum* sequence (L16996). Among the seven sequences obtained, a total of seven polymorphic sites were also identified (Table 2). Two out of the three *C. felis* sequences (OR815984) acquired in this study exhibited complete similarity to a sequence (MN394123) identified in cat specimens from Turkey. The remaining sequence (OR815983) showed a 99.08% similarity to MN394123, differing by only three bases. The two *C. viatorum* isolates obtained in this study shared a same sequence (OR815982) which exhibited identical sequences to the human-derived sequence MW014315 from China. Neither sequences of *C. baileyi* (OR815992 and OR815993) obtained here had been described, but they exhibited only minor differences, with a single base difference at 240 and 359 sites compared with MH028033, respectively.

**Table 2:** Variation at seven polymorphic sites within *SSU rRNA* gene sequences of human-derived *Cryptosporidium parvum* isolates obtained in the present study

GenBank accession no.	Nucleotide at position (relative to the start of that sequence)						
	82	203	232	283	309	311	331
OR815985	G	G	T	A	T	A	T
OR815986	G	G	T	A	T	C	T
OR815987	G	A	T	A	T	T	T
OR815988	A	G	T	A	T	T	T
OR815989	A	G	T	A	T	T	C
OR815990	A	G	T	C	T	T	T
OR815991	G	G	A	A	C	C	T

### Subtyping of *C. parvum, C. viatorum, C. felis gp60* gene

The amplification of the *gp60* gene was achieved in all 15 isolates of *C. parvum*, as well as in two out of the three isolates of *C. felis* and two isolates of *C. viatorum*. Two representative *gp60* gene sequences were observed among the 15 *C. parvum* isolates, comprising OR963532 (*n* = 14) and OR963531 (*n* = 1). The observed sequences (OR963532 and OR963531) exhibited complete similarity, reaching 100%, with the subtypes IIdA19G1 (KM199738) and IInA10 (KU852717), respectively. The presence of subtype IInA10 was identified in a boy with symptoms of diarrhea. In the present investigation, it was shown that the sequence (OR963530) of the two *C. felis* isolates exhibited a similarity of 99.81% with the sequences MW351828 (XIXa-89) and MT458682 (XIXa-E1). The sequence (OR963529) of two isolates of *C. viatorum* examined in this investigation exhibited a complete similarity of 100% to the sequence of XVaA3g (MK796004).

## Discussion

This work represents the first molecular investigation of *Cryptosporidium* among children residing in Wenzhou, China. The findings indicate that the average rate of infection was 2.1%, a rate that is comparatively lower than the combined prevalence of *Cryptosporidium* infection observed in children across China (2.9%) [14]. Variations are reported in the prevalence of *Cryptosporidium* infection among children throughout different provinces in China. As an example, the prevalence of *Cryptosporidium* infection in Henan province was recorded at 0.9% [14]. However, in Inner Mongolia, it exhibited a much higher rate of 8.9% [14]. Overall, the prevalence of *Cryptosporidium* infection among Chinese children is notably lower compared to rates observed in children from certain developing countries. For instance, Bangladesh reported a rate of 77% [18], India’s under-2-year-olds exhibited a rate of 92.4% [19], rural western Kenya’s 6 to 24-month-olds had a rate of 88.7% [20], and children under 2 years old from eight countries across Africa, Asia, and South America demonstrated an approximate rate of 65% [21]. Furthermore, when comparing China’s prevalence to certain developed countries, the rates are also lower, such as Australia’s Aboriginal children had a rate of 8.2% [22], and children with diarrhea in Switzerland had a rate of 5.5% [23]. However, reports from asymptomatic children in developed countries reveal relatively low prevalence rates, such as 0.9% in Madrid, Spain [24], 1.3% among children under 5 years old in Canada [25], and 1.3% among asymptomatic preschool children in the UK [26]. Germany, has a prevalence level similar to our study, at 2.5% [27]. These disparities could be attributed to various factors, including the environment, sanitary conditions, and living habits of different countries. We hypothesize that the relatively lower occurrence of *Cryptosporidium* infection in China can be attributed to the widespread access to safe drinking water, with approximately 90% of drinking water being boiled in the country [28]. This practice significantly reduces the risk of ingesting infectious *Cryptosporidium* oocysts, thereby mitigating the potential harm. However, it is imperative not to overlook this issue, as incidences of infection persist among Chinese children. It is noteworthy that the detection of *Cryptosporidium* in asymptomatic children also has a significant public health implications. Therefore, regular monitoring of *Cryptosporidium* infection in the population should be continued.

*Cryptosporidium* infection is well recognized as a prominent etiological factor contributing to the occurrence of diarrhea in pediatric populations [29]. In this study, the proportion of children with diarrhea was 2.5%, which was higher than non-diarrheal children. Other studies have drawn similar conclusions. For example, in Saudi Arabia, the proportion of asymptomatic children infected with *Cryptosporidium* was 4.7%, while the children with diarrhea exhibited a proportion of 32.0% [30]. In Uganda, 25.0% of diarrheal children were infected with *C. parvum*, while only 8.5% of non-diarrheal children were infected with the same pathogen [31]. This observation is present not only among pediatric populations but also among adults. The prevalence of *Cryptosporidium* infection in individuals suffering from diarrhea was notably higher compared to those without diarrhea [29]. Consequently, it is imperative to enhance the detection of *Cryptosporidium* in individuals with diarrhea, as this would facilitate a more comprehensive assessment of its impact and enable proactive clinical management of diarrhea-related complications.

This study found no statistically significant disparity in the prevalence of *Cryptosporidium* infection between boys and girls. However, it is worth noting that infection rates were somewhat higher among males in both groups. Several other studies have also shown that sex is not the main cause of the differences in *Cryptosporidium* infection rates [14, 16].

The findings of this investigation revealed that *C. parvum* was the most prevalent *Cryptosporidium* species identified in the studied population with15/22 (68.2%) of the positive findings. *C. parvum* infects an extensive range of hosts, including ungulates and wildlife, and is recognized as the primary zoonotic species affecting humans [4]. This is especially significant in rural regions where individuals frequently come into touch with livestock [5]. In this study, two subtypes were identified, including IIdA19G1 and IInA10. The IInA10 subtype was first found in China here and is presently limited to the Tanzania/Sweden population [32]. However, there is a lack of data on infected animals, making it difficult to determine the exact source of infection. There exist two distinct subtypes within family IIn, namely IInA8 and IInA10, both of which lack any documented instances of infected animals [32, 33]. It is not possible to determine whether this genotype can spread between humans and animals, so further investigation of the data is needed to clarify its host range and to complete the traceability of the source of infection. Despite the scarcity of data on *C. parvum gp60* subtypes in the population of China, the existing evidence has substantiated the ability of the IIdA19G1 subtype to infect human beings [13, 34]. Moreover, this particular subtype has been identified in children and individuals afflicted with AIDS in China [13–15, 35]. Simultaneously, it is observed that the prevalence of *C. parvum* infection in animals originating from China is extensive, with a notable abundance of *gp60* subtypes. Particularly, the IIdA19G1 subtype shows a high frequency among animals in China and demonstrates a broad geographic dispersion [34]. The subtype IIdA19G1 has been identified as a significant contributor to the rising fatality rates observed in several cryptosporidiosis outbreaks in dairy calves in China [34, 36, 37]. Hence, it is imperative to enhance the routine surveillance of individuals and animals, taking into account the aspects of animal and human health. This should be particularly emphasized for vulnerable populations such as children and those affected by AIDS.

In the present study, *C. felis* was found in three stool samples. *C. felis* is typically harbored by feline hosts and has been documented in HIV-infected individuals in Ethiopia [38], both HIV-infected and non-infected individuals in Nigeria [39], and children under the age of 5 in Kenya [40]. Research findings indicate that there is a significant likelihood of zoonotic transmission of *C. felis*. The parasite is found in other animal species, such as non-human primates, foxes, horses, and calves, in addition to cats and people, indicating the potential for zoonotic transmission [10, 41]. Furthermore, it was observed that there are five distinct subtype families of *C. felis*, namely XIXa, XIXb, XIXc, XIXd, and XIXe. Notably, two of these subtypes (XIXa and XIXb) were shown to be present in both humans and cats, providing evidence for the possibility of zoonotic transmission [10, 41]. Conversely, the other three subtypes (XXc, XIXd and XIXe) may be specific for human-to-human transmission [41]. The two *C. felis* identified in the current research belong to the XIXa subtype, indicating a risk of cat-to-human transmission. Pet owners should, therefore, be educated regarding the risk of cryptosporidiosis from their pet cats.

In this study, two children were infected with *C. viatorum*, a species originally reported in 2012 in travelers returning from India [42]. To date, *C. viatorum* has been documented in the human population throughout a multitude of nations, surpassing a total of 13 countries, including China [43–45]. The species was previously believed to only affect humans due to its infrequent occurrence in animals. However, recent research conducted in Australia and China has shown the presence of this species in wild rats [46–48]. These findings imply that *C. viatorum* has the potential to transmit between rodents and humans. Subtype partitioning supports the potential for animal-borne transmission of *C. viatorum* between rodents and humans due to the presence of its three subtypes (XVaA3g, XVaA3h, and XVcA2G1) in both humans and rodents [43]. The present finding showed that the XVaA3g was present in two children, further supporting the hypothesis that *C. viatorum* can complete reciprocal transmission between humans and rodents. To gain a more comprehensive understanding of the distribution of rodents as hosts for *C. viatorum*, it is essential to conduct screenings throughout wider geographic regions.

Although *C. baileyi* has already been found in humans, its identification of two children here is nevertheless intriguing [49]. The finding suggests the possibility of human infection from avian to *Cryptosporidium* oocysts. *C. baileyi*, which is recognized as a parasite restricted to avian species, can infect over 20 different bird hosts, including poultry, domesticated animals, and wild birds [50]. A recent study showed that the absolute infection rate of *C. baileyi* in broilers was as high as 96.1% [51]. Thus, humans coming in contact with poultry chicken or with eggs contaminated with chicken feces may have played a role in the transmission of *Cryptosporidium* infection. Additional studies are required to determine the potential of *C. baileyi* to infect humans and to understand its transmission patterns. It is anticipated that further research in this field will bring more enlightenment to public health.

## Conclusions

This work was the first molecular examination conducted on *Cryptosporidium* infection among children residing in Wenzhou, China. The mean prevalence of *Cryptosporidium* in children was found to be 2.1%. Nonetheless, it was observed that children presenting with diarrhea were more likely to have *Cryptosporidium* compared with asymptomatic children. A total of four distinct species of *Cryptosporidium* were found, namely *C. parvum*, *C. felis*, *C. viatorum* and *C. baileyi*. Among them, *C. parvum* and its subtype, IIdA19G1, have well-known zoonotic properties. Furthermore, the identification of subtypes XIXa of *C. felis* and XVaA3g of *C. viatorum* further revealed the possibility of transmission of cryptosporidiosis from animals to humans. This work was the first to document the prevalence of *C. baileyi* infection in Chinese children. This study presents compelling data on the potential origin of *Cryptosporidium* infection among children in Wenzhou, particularly emphasizing the potential transmission *via* animals, including cats, rats, and chickens. It is imperative to adopt a heightened level of vigilance in implementing efficacious preventive measures aimed at mitigating the likelihood of infection in children.

## Methods

### Sample Collection

**Fig. 1:**
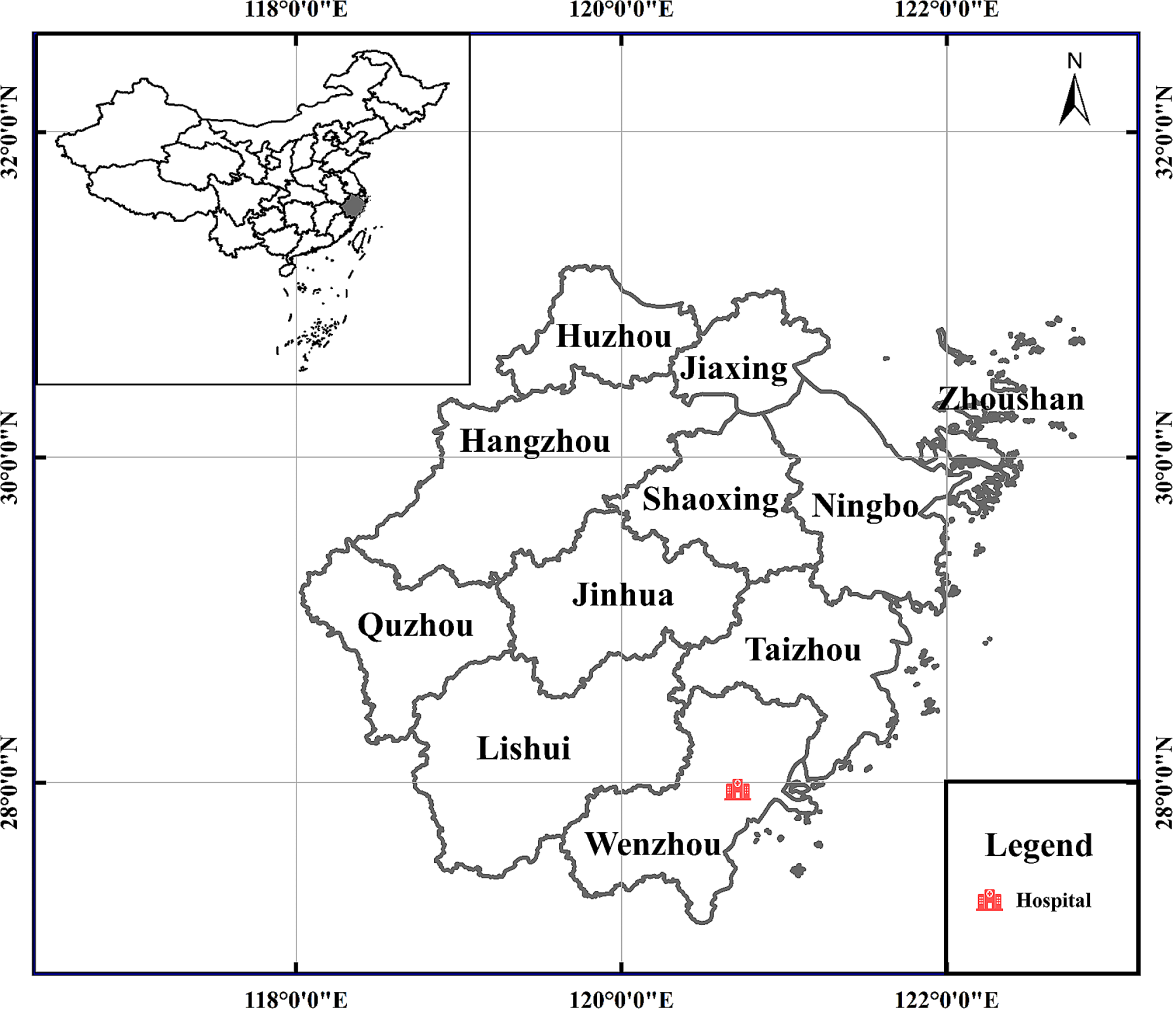
A map of the sampling hospital location in Wenzhou, China. The figure was originally designed by the authors under the software ArcGIS 10.4. The original vector diagram imported in ArcGIS was adapted from National Geomatics Center of China (http://www.ngcc.cn). The map has been modified and assembled according topermission and attribution guidelines using the softwares of Microsoft PowerPoint 2003 and Adobe Photoshop CS6.

From March 2021 to January 2022, a total of 1032 children were recruited at Yuying Children’s Hospital in Wenzhou, China (Fig. 1). Among these children, 348 were found to be asymptomatic, while 684 had symptoms of diarrhea (Table 1). The parents/guardians were guided for the collection of the fecal material in a plastic fecal collector with appropriate labeling, having the date of collection and relevant details of the patient, such as age and sex. Following the collecting process, the samples were then kept at 4 °C.

### DNA extraction and PCR amplification

The genomic DNA was obtained from fecal material weighing between 180 and 200 mg using a QIAamp DNA Stool Mini Kit (QIAgen, Hilden, Germany). following the instructions provided by the manufacturer. The presence of *Cryptosporidium* was identified in the collected DNA by the utilization of nested PCR amplification targeting a specific region of the *Cryptosporidium* genome, approximately ∼ 587 bp of the partial *SSU rRNA* gene fragment. The primers used were described previously in a study by Ryan et al. (2003) [17]. The PCR amplifications in this study were performed using TaKaRa Taq DNA Polymerase (TaKaRa Bio Inc., Tokyo, Japan). A negative control consisting of dH_2_O was included, while a positive control was prepared using DNA extracted from *C. xiaoi* obtained from goats. The isolates of *C. parvum*, *C. felis*, and *C. viatorum* were subjected to subtyping using nested PCR of the gp60 gene [9–11]. The primers and PCR program settings utilized in the current investigation are shown in Table 3. The PCR products underwent electrophoresis on a 1.5% agarose gel and were observed using a Gel Doc EZ UV-gel imaging equipment manufactured by Bio-Rad Inc. (USA). To facilitate visibility, GelRed (Biotium Inc., Hayward, CA) was used to dye the gel.

**Table 3:** The primers employed for identifying *Cryptosporidium* spp., and subtypes of *C. parvum*, *C. viatorum*, *C. felis* in the present study

Usage (s)	Primer sequence (5′ to 3′)	Fragment length (bp)	Annealing temperature (°C)	Gene	Ref
*Cryptosporidium* genus specific	18SiCF2: GACATATCATTCAAGTTTCTGAC 18SiCR2: CTGAAGGAG TAAGGAACAACC 18SiCF1: CCTATCAGCTTTAGACGGTAGG 18SiCR1: TCTAAGAATTTCACCTCTGACTG	∼ 587	58 58	SSU rRNA	[17]
Subtyping of *C. parvum*	AGP-F1: ATAGTCTCCGCTGTATTC AGP-R1: GGAAGGAACGATGTATCT AGP-F2: TCCGCTGTATTCTCAGCC AGP-R2: GCAGAGGAACCAGCATC	∼ 850	55 58	gp60	[9]
Subtyping of *C. viatorum*	CviatF2: TTCATTCTGACCCCTTCATAG CviatR5: GTCTCCTGAATCTCTGCTTACTC CviatF3: GAGATTGTCACTCATCATCGTAC CviatR8: CTACACGTAAAATAATTCGCGAC	∼ 950	55 52	gp60	[11]
Subtyping of *C. felis*	GP60CF_F1: TTTCCGTTATTGTTGCAGTTGCA GP60CF_R1: ATCGGAATCCCACCATCG AAC GP60CF_F2: GGGCGTTCTGAAGGATGT AA GP60CF_R2: CGGTGGTCTCCTCAGTCTTC	∼ 900	55 55	gp60	[10]

### Sequence and statistical analyses

All PCR products of the expected size were successfully purified using the DNA Gel Purification Kit (Sangon, Shanghai, China) and sent for a bi-directional Sanger sequencing analysis (performed by Sangon, Guangzhou, China). Sequencing was conducted using the BigDyeTerminator v3.1 Cycle Sequencing Kit (from Applied Bio systems, Carlsbad, CA, USA) on an ABI Prism 3730 XL DNA Analyzer. DNASTAR Lasergene EditSeq v7.1.0 (http://www.dnastar.com/) was employed for editing the generated sequences, while Clustal X v2.1 (http://www.clustal.org/) was utilized for aligning them with reference sequences obtained from GenBank.

The data analysis was conducted using SPSS version 22.0 (SPSS Inc., IL, USA). The chi-square test and 95% confidence intervals (CIs) were employed to compare the prevalence of *Cryptosporidium* between diarrheic and asymptomatic individuals, as well as between boys and girls. A *P*-value less than 0.05 was considered to be statistically significant.

### Nucleotide sequence accession numbers

The representative nucleotide sequences obtained in the present study were deposited in GenBank database under the following accession nos.: OR815982 to OR815993 (SSU), OR963529 to OR963532 (gp60).

## Data Availability

The representative nucleotide sequences obtained in the present study were deposited in GenBank database under the following accession nos.: OR815982 to OR815993 (SSU), OR963529 to OR963532 (gp60).

## Abbreviations

SSU rRNA: Small subunit ribosomal RNA
*gp60*: Glycoprotein 60-kDa
